# Post-traumatic stress disorder and mental health assessment of seafarers working on ocean-going vessels during the COVID-19 pandemic

**DOI:** 10.1186/s12889-022-12673-4

**Published:** 2022-02-06

**Authors:** Fereshteh Baygi, Christine Blome, Andrew Smith, Nami Mohammadian Khonsari, Arash Agoushi, Arman Maghoul, Mohammad Esmaeili-Abdar, Armita Mahdavi Gorabi, Mostafa Qorbani

**Affiliations:** 1 Research Unit of General Practice, Department of Public Health, University of Southern Denmark, Odense, Denmark; 2 Institute for Health Services Research in Dermatology and Nursing (IVDP), University Medical Center Hamburg-Eppendorf (UKE), Hamburg, Germany; 3 Centre for Occupational and Health Psychology, School of Psychology, Cardiff University, Cardiff, UK; 4 Non-communicable Diseases Research Center, Alborz University of Medical Sciences, Karaj, Iran; 5 Student Research Committee, Alborz University of Medical Sciences, Karaj, Iran; 6 Non-communicable Diseases Research Center, Endocrinology and Metabolism Population Sciences Institute, Tehran University of Medical Sciences, Tehran, Iran

**Keywords:** Seafarers, Psychological impact, COVID-19

## Abstract

**Background:**

There are increasing concerns about mental health consequences of the COVID-19 pandemic among seafarers. This study aims to assess the effects of the current global health pandemic on life satisfaction and adverse psychological outcomes among seafarers.

**Methods:**

In this cross-sectional study, 470 multinational seafarers working on board ships of two international shipping companies were assessed. Mental health outcomes were assessed by the general anxiety disorder (GAD-7) questionnaire, post-traumatic stress disorder (PTSD-8) questionnaire, and patient health questionnaire (PHQ-9) depressive severity score. Multivariate logistic regression was used to determine the association of demographic and work-related variables with mental health outcomes.

**Results:**

Overall, 439 out of 470 invited seafarers with a mean age of 34.5 (SD: 8.05) years participated in this study (participation rate: 93.4%). The prevalence of anxiety, depressive, and post-traumatic stress symptoms was 12.4, 14.1, and 37.3%, respectively. In the multivariate model, the current vessel’s signing duration was directly associated with the odds of depressive and intrusion symptoms. Moreover, the duration of work per week was inversely associated with hyper-vigilance and avoidance. Also, non-officers, compared to officers, experienced significantly lower anxiety and depressive symptoms, hyper-vigilance, and avoidance.

**Conclusion:**

The present study revealed a high prevalence of mental health problems among seafarers during the COVID-19 pandemic. We recommend that more evidence is generated regarding psychosocial health issues for this vulnerable occupation.

## Background

The shipping industry plays a vital role in international trade and global supply chains [1]. Even in extraordinary circumstances like the global pandemic of COVID-19, goods and services are to be exchanged across international borders [2]. Thus, seafarers have a critical function in keeping the wheels of the world economy moving.

Studies have revealed the impact of a previous health emergency - Severe Acute Respiratory Syndrome (SARS) - on the general population’s mental health [3, 4]. It is also evident that the current COVID-19 pandemic could affect mental health and wellbeing now and in the coming years [5]. A recent rapid review revealed quarantine-related stressors, including fear of infection, frustration, inadequate information, and boredom, which may result in post-traumatic stress symptoms and anger [6]. However, the safety of the workplace should not go unnoticed since risk assessment, and preventive action within the workplace can affect the workers’ mental health [7, 8].

Previous studies in the maritime setting found that, even under normal conditions, seafarers’ health is affected by their living and working circumstances on board [9]. Inability to leave the workplace, living and working in the same environment, and restricted contact with family members have been mentioned as challenges of working at sea [10–13]. During the current health emergency, many port authorities prohibit seafarers from disembarking upon arrival at the port to stop the epidemic spread of COVID-19 [14]. A recently published umbrella review on mental health outcomes of quarantine and isolation for infection prevention revealed that depression, post-traumatic stress symptoms, and anxiety disorders are highly prevalent among people with physical isolation [15]. So, Prolonged stay at sea and extended periods of social isolation due to port restrictions might bind to heighten mental health problems among seafarers.

According to our knowledge, resources for mental health services at sea are generally limited [16, 17]. In order to address a shortage of service delivery, potential risk factors affecting the mental health of seafarers during the COVID-19 pandemic should be assessed. Especially since the existing data regarding seafarers, even before the pandemic, are highly controversial and no precise data on the prevalence of mental issues exist [18]. Therefore, this study aims to identify risk factors that can affect seafarers’ mental health in this extraordinary situation. Findings from this study will contribute to the United Nations Sustainable Development Goals (Goal number 3: Good Health and Well-being) and may help improve seafarers’ health and wellbeing in the future.

## Materials and methods

### Study design

In July 2020, a cross-sectional study was performed among two international shipping companies seafarers. Four hundred seventy multi-nationality seafarers were selected via the convenience sampling method. Because of the descriptive nature of the study, sample size calculation was not done.

### Data collection

Data were obtained with self-administrated questionnaires, including demographic and work-related characteristics such as age, marital status, position and duties on the ship, working days and hours, and ship characteristics. The standardized and validated mental health questionnaires, including General Anxiety Disorder (GAD-7) [19], Posttraumatic Stress Disorder (PTSD-8) [20], and Patient Health Questionnaire (PHQ-9) [21], were used to assess different aspects of psychological well-being. Since all participants could read, understand, and speak English fluently, the English version of all questioners was used for this study.

PTSD-8 is a short screening tool consisting of 8 items that assess possible post-traumatic stress disorder symptoms in the following domains: intrusion, avoidance, and hyper-vigilance [22]. Items are rated on a four-point Likert scale (1 = not at all, 4 = very often). At least one item with a score of 3 or higher within each PTSD domain was considered a cutoff score indicating possible PTSD [22].

GAD-7 is a seven-item questionnaire with high sensitivity in detecting anxiety – panic, social anxiety, and post-traumatic stress disorders [20]. Items are rated from “0 = not at all to 3 = nearly every day”. Score of 5, 10 and 15 were considered as the cut-off points for mild, moderate and sever anxiety [20].

PHQ-9 is a self-assessment tool for depression and consists of nine items. A four-point scale ranging from “0 = nearly every day” to “3 = not at all” is used to show the depressive symptoms and declines in interest during the last 2 weeks [23].

### Statistical analysis

Data were analyzed using SPSS (Statistical Package for the Social Sciences software, version 16). The normal distribution of continuous variables was assessed using the Kolmogorov-Smirnov test, and due to normal distribution, continuous variables are expressed as mean and standard deviation (SD). Categorical variables are expressed as frequency and percentage. The prevalence of psychiatric symptoms was reported with a 95% confidence interval (CI). Independent t-tests were used to compare the characteristics of continuous demographic and work-related characteristics (age, working hours, duration of signing on the current vessel) across psychiatric symptoms and association of categorical demographic and work-related characteristics across PTSD subscales, GAD, and PHQ scores using Chi-square tests. Correlations between GAD, PHQ, and PTSD were assessed using Pearson’s correlation coefficient.

Univariate and multivariate logistic regression analyses were used to determine the association of demographic and work factors with the aforementioned psychological symptoms. Variables with a *p*-value< 0.1 in the univariate model were included in the multivariate model. Logistic regression analysis results were reported as odds ratio (OR) and 95% CI. A two-tailed *p*-value below 0.05 was considered statistically significant.

## Results

From 470 invited seafarers, 439 of them filled out and returned the questionnaires (participation rate: 97.6%). Mean age of seafarers was 34.5 (SD: 8.05). Most of the seafarers were Indian (77.7%) and married (67.9%). 46.8% were officers, and 51.4% were day shift personnel. 51.8 and 38.2% of participants worked on deck and in the engine room, respectively. 57.6% of the ships were crude oil tankers.

Prevalence (95% CI) of intrusion, hypervigilance and avoidance was 25.1% (95% CI: 21.1–29.4), 22.2% (95% CI: 18.4–26.4), and 21.1% (95% CI: 17.4–25.2) respectively. Overall, 37.3% (95% CI: 32.8–42.0) of seafarers had disruptions within at least one of the PTSD subscales, and 11.8% (95% CI: 8.9–15.2) had disruptions within all three domains of PTSD.

**Fig. 1:**
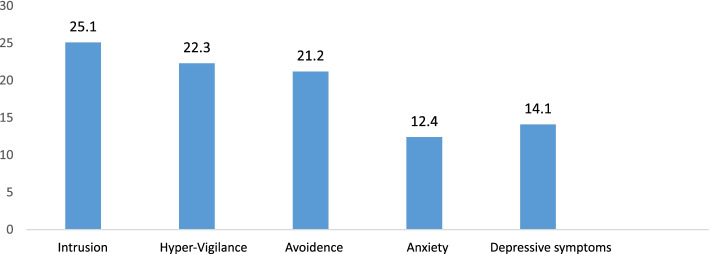
Prevalence of post-traumatic stress symptoms, anxiety, and depressive symptoms among seafarers. Despite depressive and anxiety symptoms having a high prevalence, as illustrated in the figure, the prevalence of post-traumatic stress symptoms (intrusion, avoidance, hypervigilance) was higher in comparison with anxiety and depressive symptoms

Anxiety symptoms were prevalent in 12.4% (95% CI: 9.5–15.9) of seafarers. Depressive symptoms were observed in 14.1% (95% CI 11.0–17.7) of seafarers, respectively (Fig.1).

The prevalence of depressive symptoms was significantly greater among officers, married personnel, and those with a higher average signing on in the current vessel (during the COVID-19 period). Other demographic and work-related variables did not differ significantly across anxiety and depressive symptoms (*p* > 0,05) (Table 1).

**Table 1:** Association of demographic and work-related variables with PTSD subscales, anxiety and depressive symptoms among seafarers

Psychiatric symptoms	categories	Age (year) 1	AWH*	ASIV (days)**	Marital status		Work status		Duties			Position	
					Single	Married	Day	Shift	Deck	Engine room	Service	Officer	Non-officer
**Anxiety**	**Yes**	**35.98 ± 8.13**	**69.61 ± 11.75**	**115.73 ± 63.13**	**11 (8)**	**44 (14.7)**	**28 (13)**	**25 (12)**	**34 (15)**	**19 (11.5)**	**2 (4.7)**	**38 (19)**	**17 (7.3)**
	**No**	**34.28 ± 8.03**	**66.96 ± 9.21**	**124.18 ± 60.07**	**130 (92)**	**254 (85.3)**	**190 (87)**	**186 (88)**	**191 (85)**	**147 (88.5)**	**41 (95.3)**	**163 (71)**	**216 (92.7)**
	***p*-value**	**0.14**	**0.055**	**0.35**	0.04*		**0.75**		**0.25**			< 0.001*	
**Depressive symptoms**	**Yes**	**35.48 ± 7.54**	**66.46 ± 11.90**	**133.97 ± 62.02**	**13 (9)**	**49 (16.5)**	**34 (15.6)**	**24 (11.4)**	**38 (17)**	**21 (12.7)**	**3 (7)**	**43 (21.4)**	**18 (7.7)**
	**No**	**34.33 ± 8.13**	**67.43 ± 9.17**	**114.29 ± 63.33**	**128 (91)**	**249 (83.5)**	**184 (84.4)**	**187 (88.6)**	**187 (83)**	**145 (87.3)**	**40 (93)**	**158 (78.6)**	**215 (92.3)**
	***p*-value**	**0.29**	**0.46**	0.04*	0.04*		**0.2**		**0.25**			< 0.001*	
**Intrusion**	**Yes**	**35.44 ± 8.09**	**66.39 ± 9.65**	**128.63 ± 50.11**	**29 (20.6)**	**81 (27.2)**	**55 (25)**	**50 (24)**	**54 (23)**	**43 (25.9)**	**13 (26.6)**	**70 (35)**	**39 (17)**
	**No**	**34.18 ± 8.03**	**67.60 ± 9.57**	**112.86 ± 66.01**	**112 (79.4)**	**217 (72.8)**	**163 (75)**	**161 (76)**	**171 (76)**	**123 (74.1)**	**36 (73.4)**	**131 (65)**	**194 (83)**
	***p*-value**	**0.15**	**0.25**	0.02*	**0.13**		**0.71**		**0.83**			0.001 > *	
**Hyper-vigilance**	**Yes**	**36.12 ± 8.42**	**64.02 ± 9.95**	**124.57 ± 55.43**	**20 (14.2)**	**78 (26.2)**	**55 (25)**	**39 (28.5)**	**45 (20)**	**40 (24)**	**16 (32.7)**	**56 (28)**	**41 (17.6)**
	**No**	**34.03 ± 7.89**	**68.24 ± 9.29**	**114.58 ± 64.58**	**121 (85.8)**	**220 (73.8)**	**163 (75)**	**172 (81.5)**	**180 (80)**	**126 (76)**	**33 (67.3)**	**145 (72)**	**192 (82.4)**
	***p*-value**	0.02*	0.001 > *	**0.16**	0.005*		**0.09**		**0.25**			0.01*	
**Avoidance**	**Yes**	**35.56 ± 8.11**	**64.88 ± 9.85**	**123.23 ± 47.73**	**24 (17)**	**69 (23)**	**47 (21.5)**	**41 (19.5)**	**44 (19.5)**	**43 (25.9)**	**6 (12.3)**	**55 (27.4)**	**36 (15.5)**
	**No**	**34.21 ± 8.02**	**67.95 ± 9.43**	**115.08 ± 66.13**	**117 (83)**	**229 (77)**	**171 (78.5)**	**170 (80.5)**	**181 (80.5)**	**123 (74.1)**	**43(87.7)**	**146 (72.6)**	**197 (84.5)**
	***p*-value**	**0.14**	0.006*	**0.26**	**0.14**		**0.58**		**0.17**			0.002*	

Anxiety and depressive symptoms were assessed using General Anxiety Disorder-7 and Patient Health Questionnaire-9 respectively

^*^Average work hours per week

^**^Average signing on in the current vessel (months)

The association of demographic and work-related variables with PTSD subscales is presented in Table 1. Hyper-vigilance score was significantly higher within married crew members in comparison to the singles. The intrusion score was significantly higher among those with a longer stay duration (during the COVID-19) on board in the current vessel. Hyper-vigilance and avoidance score were significantly higher among those with less mean work per week. All PTSD subscale scores were significantly higher among officers in comparison to other crew members.

Correlation between different psychosocial variables in seafarers is presented in Table 2. The correlation between PTSD subscales, GAD, and PHQ-9 score was statistically significant.

**Table 2:** Pearson’s correlation between age, work time during corona (in hours), GAD anxiety severity score, PHQ-9 depressive symptoms severity score and PTSD subscales among seafarers

	Age	work time during corona (in hours)	PHQ-9 depressive severity score	GAD-7 score	Intrusion	Avoidance	Vigilance
**Age**	**1**	**−0.056**	**0.039**	**0.073**	0.096*	0.108*	0.124*
**work time during corona (in hours)**	**−0.056**	**1**	0.116*	0.104*	0.106*	**0.092**	**0.046**
**PHQ-9 depression severity score**	**0.039**	0.116*	**1**	0.761*	0.543*	0.398*	0.379*
**GAD-7 score**	**0.073**	0.104*	0.761*	**1**	0.550*	0.418*	0.397*
**Intrusion**	0.096*	0.106*	0.543*	0.550*	**1**	0.669*	0.678*
**Avoidance**	0.108*	**0.092**	0.398*	0.418*	0.669*	**1**	0.577*
**Vigilance**	0.124*	**0.046**	0.379*	0.397*	0.678*	0.577*	**1**

^*^statistically significant (*p*-value < 0.05)

The association of demographic and work-related variables with psychiatric symptoms in logistic regression analysis is presented in Table 3. In the multivariate (adjusted) model, per additional month of signing in during the COVID-19 pandemic in the current vessel, the odds of intrusion (OR: 1.16; 95% CI: 1.03–1.31, *p* < 0.01) and depressive symptoms (OR: 1.15; 95% CI: 1.01–1.31, *p* < 0.01) increased by approximately 15%. Moreover, by increasing each hour’s work per week, the odds of hyper-vigilance and avoidance decreased significantly (OR: 0.96; 95% CI: 0.94–0.99, *p* < 0.01).

**Table 3:** Association of demographic and work-related variables with psychiatric symptoms and PHQ-9 depressive symptoms severity score in logistic regression analysis among seafarers

		Age (year) 1	AWH (hour)^**a**^	ASIV (months)^**b**^	Marital statu	Work status	Duties		Position
					Single /Married	Day/Shift	Deck /Engine room	Deck /Service	Officer/Non-Officer
**Anxiety**	**Model I**	**1.02 (0.99–1.06)**	**1 (0.99–1)**	**1.06 (0.93–1.22)**	**2.04 (1.02–4.09)**^**a**^	**0.91 (0.51–1.62)**	**0.72 (0.39–1.32)**	**0.27 (0.06–1.18)**	**0.33 (0.18–0.61)**^**a**^
	**Model II**	**(−)**	**(−)**	**(−)**	**2.22 (0.90–5.45)**	**(−)**	**(−)**	**(−)**	**0.30 (0.15–0.61)**^**a**^
**Depressive symptoms**	**Model I**	**1.01 (0.98–1.05)**	**0.98 (0.96–1.01)**	**1.15 (1.01–1.31)**^**a**^	**1.93 (1.01–3.70)**^**a**^	**0.69 (0.39–1.21)**	**0.71 (0.40–1.26)**	**0.36 (0.10–1.25)**	**0.3 (0.17–0.55)**^**a**^
	**Model II**	**(−)**	**(−)**	**1.16 (1.01–1.34)**^**a**^	**1.88 (0.82–4.31)**	**(−)**	**(−)**	**(−)**	**0.26 (0.13–0.52)**^**a**^
**Intrusion**	**Model I**	**1.01 (0.99–1.04)**	**0.98 (0.96–1)**	**1.13 (1.01–1.26)**^**a**^	**1.44 (0.89–2.33)**	**0.92 (0.59–1.43)**	**1.10 (0.69–1.75)**	**1.22 (0.58–2.55)**	**0.37 (0.24–0.59)**^**a**^
	**Model II**	**(−)**	**(−)**	**1.16 (1.03–1.30)**^**a**^	**(−)**	**(−)**	**(−)**	**(−)**	**0.31 (0.18–0.53)**
**Hyper-vigilance**	**Model I**	**1.03 (1–1.06)**^**a**^	**0.95 (0.93–0.97)**^**a**^	**1.08 (0.96–1.20)**	**2.14 (1.25–3.67)**^**a**^	**0.67 (0.42–1.06)**	**1.27 (0.78–2.05)**	**1.73 (0.83–3.59)**	**0.55 (0.35–0.87)**^**a**^
	**Model II**	**1 (0.96–1.03)**	**0.94 (0.92–0.97)**^**a**^	**(−)**	**1.81 (0.91–3.57)**	**(−)**	**(−)**	**(−)**	**0.49 (0.29–0.85)**^**a**^
**Avoidance**	**Model I**	**1.02 (0.99–1.04)**	**0.96 (0.94–0.99)**^**a**^	**1.06 (0.95–1.19)**	**1.46 (0.87–2.45)**	**0.87 (0.54–1.40)**^**a**^	**1.43 (0.89–2.32)**	**0.54 (0.2–1.45)**	**0.48 (0.30–0.77)**^**a**^
	**Model II**	**(−)**	**0.96 (0.94–0.99)**^**a**^	**(−)**	**(−)**	**(−)**	**(−)**	**(−)**	**0.57 (0.34–0.97)**^**a**^

Anxiety and depressive symptoms were assessed using General Anxiety Disorder-7 and Patient Health Questionnaire-9 respectively

Model I: crude model

Model II: adjusted for variables which had *p*-value< 0.1 in the crude model

^a^Average work hours per week

^b^Average signing on in the current vessel (months)

In the adjusted model, non-officer crews experienced significantly lower anxiety symptoms (OR: 0.30; 95% CI: 0.15–0.61, *p* < 0.001), depressive symptoms (OR: 0.3; 95% CI: 0.17–0.55, *p* < 0.01), hyper-vigilance (OR: 0.49; 95% CI: 0.29–0.85, *p* < 0.01) and avoidance (OR: 0.57;95% CI: 0.34–0.97, *p* < 0.01) as compared to officer crews.

## Discussion

Although several studies in different study groups (e.g., general population, health care providers, patients) have assessed the mental health outcomes during the COVID-19 pandemic [24–32], to the best of our knowledge, this is the first study that assesses mental health outcomes of seafarers during the COVID-19 pandemic. Therefore, this study provides a snapshot of the psychological status of seafarers under the shadow of the COVID-19 pandemic.

According to our study, the prevalence of anxiety symptoms, depressive symptoms, and PTSD was 12.4, 14.1, and 37.3%, respectively. A recently published meta-analysis on the impact of coronavirus syndrome (MERS vs. SARS vs. COVID-19) on the mental and physical health of health care workers (HCW) revealed that the prevalence of anxiety symptom features, depressive symptoms, and PTSD was 29.0, 26.3, and 20.7%, respectively [33]. The lower observed prevalence of psychological outcomes among seafarers compared to HCW may be related to the unsimilar workplace setting. HCW are working in the frontline of the health care system, and they are in close contact with COVID-19 infected people. So, the extent and type of the stressors or mental health issues in such an occupation are entirely different with seafarers working in the isolated workplace.

A cross-sectional study conducted on the young Chinese population during COVID-19 showed that 40.4% of the studied population was prone to psychological problems; the prevalence of PTSD was 14.4% [34]. Although a similar questionnaire has been used in our study and Chinese youth study, but the prevalence rate of PTSD among seafarers was much higher than Chinese people. This discordant finding may be justified by different study groups and time of PTSD measurement. Our study was conducted 4 months after the COVID-19, while the Chinese youth study was performed just 2 weeks after the emergency.

A comparison study among people affected and unaffected by quarantine during the COVID-19 pandemic showed a higher prevalence of depressive symptoms and anxiety symptoms among the affected group, which might be related to long-term social isolation. Also, the overall prevalence of anxiety symptoms and depressive symptoms in the studied population was 8.3 and 14.6%, respectively [35]. The findings of our study are in line with mentioned study, although the settings are different. We think the isolated nature of the workplace at sea might be the possible reason for such similarity of the prevalence rate of the two studies.

Our study revealed that the prevalence of depressive symptoms among married officers who had been on board for a more extended period during the COVID-19 pandemic was higher than the other crew members. Long periods of separation from loved ones, especially kids, might be a reason for such finding among married officers. A recently published study about COVID-19 in maritime setting revealed that apart from long term isolation, additional factors related to current health emergency including pressure to get home from family members, concern about family members’ health in vulnerable cities, limited medical facilities, lack of awareness, and less access to medical care ashore- because of COVID-19 port restrictions- might also have adverse effects on the psychological status of people at sea [36]. Although this study fails to provide seafarers’ perspectives regarding the current extra-ordinary situation at se, we think that in our study, such kinds of factors might also affect seafarers’ mental health status.

In our study, the general anxiety score was higher among officers compared to non-officers. We know that seafarers have a high potential to be infected with COVID-19 when traveling from home to ship, ship to home, or visiting port facilities [36]. Officers are in contact with the port during load and discharge. Also, they are responsible for the safety of the crew members on board. We think such close contact with port authorities, together with the responsibility for providing the safety of people on board, might cause more stress and fear of being infected by COVID-19. Consequently, such pressure may induce higher anxiety symptoms level.

Another study also suggests that the mental health of people, especially vulnerable ones, can be affected by several psychological factors such as fear, stigma, and lack of awareness [29]. We assume that ship-specific stress situations, together with lack of awareness about the COVID-19 situation and less access to medical care ashore, might cause fear and uncertainty among seafarers. So, these factors might be the main reasons for such prevalence of anxiety symptoms and depressive symptoms among seafarers, especially in married officers. They, therefore, require particular attention with regard to psychosocial health issues during the recent global health emergency.

The current study also revealed that the odds of intrusion and depressive symptoms significantly was higher among seafarers with a more extended stay at sea during COVID-19, which supports the assumption that the symptoms are actually caused by the difficult situation on sea. This might be connected to working hard, dealing with a problematic situation, and uncertainty about the current situation and getting back home. Further studies are suggested to explore the main reasons for such findings.

We found that the odds of hyper-vigilance and avoidance decreased by increasing working hours per week. A possible reason would be that seafarers may blunt or neglect their emotions through work or sometimes by overworking. Although the study’s cross-sectional nature hampered the evaluation of causal relationships, further studies are needed to evaluate the causal relationship of mental health issues.

We found that officers experienced more anxiety symptoms, depressive symptoms, hyper-vigilance, and avoidance. Because officers are the ones who should provide a safe workplace for all crew members in such dangerous situations, also, they are physically more in touch with port authorities. Nonetheless, further studies on seafarers are needed since the data regarding seafarers, even prior to the pandemic, is controversial. Some studies have reported a low level of depression and mental issues among seafarers compared to the normal population [37, 38]. Whereas others have found a prevalence of suicidal thoughts among seafarers, as high as 35% [39].

### Limitations and strengths of the study

The cross-sectional nature of the study is the main limitation of the current work. Also, due to a lack of studies on the prevalence of the psychological issues reported here before the pandemic situation, it was challenging to determine whether the extent of psychological issues among seafarers has increased during the COVID-19 outbreak. Furthermore, the lack of data regarding prior psychiatric illness in participants was one of our limitations.

Besides, all limitations connected with self-report measures might affect the results of our study. The strength of the study is that this is the first study with a large sample size in seafarers to address the psychological status of this study group during the COVID-19 outbreak by using validated instruments.

## Conclusion

Our findings revealed the high prevalence of anxiety symptoms, depressive symptoms, PTSD among seafarers of ocean-going vessels during COVID-19. Also, a higher prevalence of anxiety symptoms, depressive symptoms, hyper-vigilance, and avoidance was observed among officers compared to non-officers. Although seafarers, as key workers have faced many unforeseen problems during the pandemic and even several suicides, have been reported on board ships [36], apart from the current study, there has been no study to address mental health issues during the COVID-19 pandemic among this hard-to-reach group. So, we would like to encourage researchers to do more research in order to cover all aspects of the psychological health of seafarers during COVID-19, then the stockholders in maritime setting to take action regarding mental health issues of seafarers at sea.

## Data Availability

The datasets generated and/or analyzed during the current study are not publicly available due the confidential policy of the shipping companies, but are available from the corresponding author on reasonable request.
